# Complete Genome Sequence of a Thin-Sheath Mutant of the Phototropic Cyanobacterium *Calothrix* sp. Strain PCC 7716

**DOI:** 10.1128/MRA.00947-21

**Published:** 2021-12-02

**Authors:** Yuu Hirose, Mitsunori Katayama

**Affiliations:** a Department of Applied Chemistry and Life Science, Toyohashi University of Technology, Toyohashi, Aichi, Japan; b College of Industrial Technology, Nihon University, Narashino, Chiba, Japan; University of Delaware

## Abstract

*Calothrix* sp. strain PCC 7716 is a filamentous cyanobacterium whose morphology is tapered, with basal-apical polarity. The apical filament shows positive phototropism toward white light or blue light. To elucidate the molecular basis of the phototropism, we determined the complete genome sequence of a spontaneous mutant of this strain that has a thin sheath and is suitable for genomic DNA extraction.

## ANNOUNCEMENT

*Calothrix* sp. strain PCC 7716 has a tapered filament with basal-apical polarity and can develop heterocysts, specialized cells for nitrogen fixation, at the base of the filament (1). *Calothrix* sp. strain PCC 7716 can reorient the direction of growth of the apical filaments toward incident light, which is the capacity called phototropism (2). To identify the genes involved in the regulation of phototropism, we obtained the wild-type strain PCC 7716 from the Pasteur Culture collection of Cyanobacteria (PCC) and attempted random gene knockout using the Tn*5* transposon in the conjugal plasmid pRL1058 (3). We did not succeed in obtaining Tn*5*-integrated clones, but we did obtain clones that transiently showed kanamycin resistance. From these clones, we obtained a spontaneous mutant (designated substrain S6) that retains phototropism but has a thin sheath and is susceptible to lysis by lysozyme, which is suitable for DNA extraction without physical disruption of the cells. We recently determined the draft genome sequence of another phototrophic cyanobacterium, *Rivularia* sp. strain IAM M-261 (4), but no genomic information is available for the PCC 7716 strain.

Here, we performed whole-genome sequencing of the S6 substrain of PCC 7716 using the MiSeq (Illumina) platform. Cells were grown at 30°C on solid BG11 medium (1) supplemented with 1% agar under an irradiance of 30 μmol photons m^−2^ s^−1^ provided by fluorescent lamps. The cells were scraped off and subjected to DNA extraction using the Wizard genomic DNA purification kit (Promega) and further purification using a Genomic-tip 20/G (Qiagen). Paired-end libraries with ∼800-bp insert size were prepared using the TruSeq DNA PCR-free library preparation kit (Illumina). Mate pair libraries with ∼8-kbp insert size were prepared using the Nextera mate pair sample preparation kit (Illumina). Each 300-bp end of the libraries was sequenced on a MiSeq sequencer with the MiSeq reagent kit v3 (600 cycles; Illumina). Base calling and demultiplexing of the reads were performed using Real-Time Analysis v1.18.54 and MiSeq Control Software v2.6.21. Correction of sequence errors based on 17-mer frequency and removal of the junction sequence of the mate pair reads were performed using ShortReadManager (5). All tools were run with default parameters unless otherwise specified.

A total of 1.23 million paired-end reads (total of 307 Mbp) and 0.78 million mate pair reads (total of 121 Mbp), corresponding to 35× coverage of the genome of the PCC 7716 strain, were assembled using Newbler v2.9 (Roche); this yielded 21 scaffolds (>2 kbp) and 246 contigs (>500 bp), with a contig *N*_50_ of 129 kbp. Gap sequences between contigs were determined *in silico* using the GenoFinisher and AceFileViewer programs as described previously (6–10), which support unraveling of the connections of unplaced repeat sequences in the gaps. We successfully determined the complete genome sequence of the S6 substrain, which consists of a circular chromosome and nine plasmids (total genome size, 12,367,066 bp). A total of 10,787 coding sequences, 10 rRNAs, 66 tRNAs, and 26 CRISPR genes were predicted and annotated using the DFAST pipeline (11). The GC content and coding proportion were calculated as 38.4% and 81.9%, respectively. The axenicity of the assembly was checked using CheckM, with the following results: completeness, 98.78%; contamination, 0.95%; strain heterogeneity, 0.0% (12). The complete genome sequence of *Calothrix* sp. strain PCC 7716 will provide new clues to the molecular basis for the regulatory mechanism of phototropism.

### Data availability.

The complete genome sequence of *Calothrix* sp. strain PCC 7716 was deposited in the DNA Data Bank of Japan (DDBJ) under accession numbers AP025018 to AP025027. Raw sequence reads used for the assembly were deposited in the DDBJ Sequence Read Archive (DRA) under accession number DRA012531.
